# Dr. Lorenzo Burrows

**Published:** 1918-11

**Authors:** 


**Dr. Lorenzo Burrows** of Buffalo died in France on September 17th of bronchial pneumonia. Dr. Burrows enlisted in the Buffalo base hospital unit 23, in August, 1917, and sailed for France in November of the same year. He served with the rank of captain, but it has been stated, though not officially, that he was very recently promoted to colonel. Dr. Burrows was a graduate of the College of Physicians and Surgeons at New York City, and had for many years been a prominent

Buffalo oculist. He had distinguished himself as a philanthropist and among all medical societies. Dr. Burrows had been for some time previous to his departure a member of the staff of the General Hospital. He is lamented by a host of friends and professional associates.

